# Association of height with peripheral arterial disease in type 2 diabetes

**DOI:** 10.1007/s40618-014-0129-y

**Published:** 2014-07-20

**Authors:** Xiuli Fu, Shi Zhao, Hong Mao, Zhongjing Wang, Lin Zhou

**Affiliations:** Department of Endocrinology, The Central Hospital of Wuhan, Tongji Medical College, Huazhong University of Science and Technology, Wuhan, 430061 Hubei China

**Keywords:** Diabetes mellitus, Height, Peripheral arterial disease

## Abstract

**Objective:**

To investigate whether height is associated with peripheral arterial disease (PAD) in patients with type 2 diabetes.

**Research design and methods:**

This was an observational study performed in 4,528 Chinese patients with type 2 diabetes. Anthropometric measures and the ankle-brachial index (ABI) were performed on each subject. PAD was defined as those patients with a history of revascularization or amputation due to ischemia, or an ABI <0.9.

**Results:**

A total of 23.3 % of T2DM patients had PAD (men 22.9 % and women 23.7 %). The mean age and height were 57.8 ± 12.5 years and 170.5 cm for men, and 60.0 ± 11.7 years and 158.9 cm for women, respectively. The ABI and frequency of PAD were higher with decreasing height quartiles. An inverse association was observed between height- and gender-adjusted risk of PAD. This relationship remained unchanged following further adjustment for potential confounders. Subjects in the shortest stature group had of 1.174 times higher risk of PAD for men and 1.143 times for women, compared with those in the tallest stature group. The multivariate adjusted hazard ratios (95 % CI) of PAD for a 10-cm height increase were 0.85 (95 % CI 0.78–0.94).

**Conclusion:**

A short stature seems to be associated with higher risk of PAD in Chinese diabetic patients. However, the cross-sectional nature of the study limits conclusions regarding the direction or causality. Further longitudinal study is warranted in this and other ethnic groups.

Height attained in adulthood is a phenotypic expression of genetic load that is influenced by various environmental and socioeconomic variables in the perinatal period and during the growth period. However, an inverse association between height and all-cause mortality was observed from a cohort study of 386,627 middle-aged South Korean male civil servants, even after adjustment for socioeconomic position and major behavioral risk factors [1]. Several prospective studies have demonstrated that height is inversely associated with incidence of or mortality from cardiovascular disease (CAD) and cerebrovascular disease (CVD) [2, 3]. Inverse linear associations were observed between height and coronary heart disease (CHD), stroke, CVD, injury and total mortality among a total of 510,800 participants with 21,623 deaths [4]. Even after correcting for conventional cardiovascular risk factors, taller individuals have more favorable central hemodynamics and reduced evidence of coronary artery disease compared with shorter individuals [5].

As an indicator of multifocal atherosclerosis, peripheral arterial disease (PAD) is emerging as an important aid in risk stratification of patients with CAD or CVD. Studies have observed that lower-extremity amputations are much less common among Taiwanese people of shorter stature than among white people who are much taller. Similarly, low procedure rates have been found in other Asian populations [6]. The reason for the difference in rates between ethnic groups in unknown, but height may be a factor. A strong and statistically significant association was revealed between height and risk of lower-extremity amputation among diabetic patients [7].

PAD is characterized by narrowing or occlusion of the arteries resulting in gradual reduction of blood supply to the limbs. Among diabetic patients, PAD is common which causes suffering and high costs by ulcerations and chronic pain as well as a major risk factor for need of limb amputations. However, it remains unknown whether height affects PAD in type 2 diabetic patients (T2DM). In this context, our aim was to study the relationship between height and PAD in T2DM.

## Methods

### Subjects and methods

Our cross-sectional study included 4,528 T2DM patients with a diagnosis of diabetes according to American Diabetes Association criteria (2005). From March 2011 to October 2013, we consecutively recruited patients who were visiting the Central Hospital of Wuhan for treatment of diabetes. All participants signed consent forms, and the ethics committee of the Central Hospital of Wuhan approved the study.

Height, weight, waist circumference (WC) and hip circumference (HC) were measured for all subjects while they were wearing light clothing and not wearing shoes. Height and WC were measured to the nearest 1 mm using a stadiometer and a metal anthropometric tape, respectively. BMI was calculated as weight divided by the square of height in meters. WC was measured at the level of the umbilicus and HC at the widest girth of the hip. Waist–hip ratios (WHpR) were calculated as WC divided by HC.

Systolic blood pressure (SBP) and diastolic blood pressure (DBP) were measured using a standard mercury sphygmomanometer. After 10 min of rest in the seated position, two readings were taken on the right arm with 5 min between each recording. The mean of the two values was used for analysis. Hypertension was defined as an SBP of 140 mmHg or higher or a DBP of 90 mm Hg or higher or current use of antihypertensive treatment. Participants were defined as current cigarette smokers if they reported smoking at least one cigarette per day over the previous year.

Fasting plasma glucose, HDL cholesterol, LDL cholesterol, triglycerides and total cholesterol were measured after an overnight fast. Dyslipidemia was defined according to Third Report of the Expert Panel on Detection, Evaluation, and Treatment of High Blood Cholesterol in Adults criteria or current treatment with statins. HbA_1c_ was determined by high-performance liquid chromatography. Urine albumin and creatinine in the morning urine sample were analyzed with a biochemical autoanalyzer. Albuminuria was determined by the urinary albumin–creatinine ratio (UACR). Estimated glomerular filtration rate (eGFR) was calculated on the basis of serum creatinine level, with the expression of the Modification of Diet in Renal Disease Study (MDRD) prediction equation for standardized serum creatinine [8].

The ankle-brachial index (ABI) was measured with an automatic device that incorporates a sphygmomanometer and two-way Doppler with an 8-MHZ probe, strictly following the procedure. Briefly, after resting for 5 min in the supine decubitus position, systolic BP was measured in both arms and the highest value was selected for calculation of the ABI (denominator). The systolic BP of the posterior tibial artery was then measured in each leg, and the highest value (whether tibial or pedal) was taken as reference for calculating the individual ABI of each leg (numerator). The ABI of both the left and right legs was recorded. For the definition of PAD the lower of the two values was taken. Normal values of ABI were considered between 0.9 and 1.3. PAD was defined as those patients with a history of revascularization or amputation due to ischemia, or an ABI <0.9.

### Statistical analysis

Continuous variables were reported as mean ± SD, and categorical factors were reported as percentages. All the continuous variables were normally distributed with homogenous variances between groups except for triglyceride, which was log transformed before entering into the model. The intergroup comparisons were performed using a one-way ANOVA test followed by a Scheffe post hoc test. Comparisons of the prevalence of PAD were made by means of a *x* ^2^ test. To estimate the odds ratio (OR) of PAD in each quartile, logistic regression was performed, and the highest quartile was used as reference category. Multivariate adjusted OR was presented with 95 % CIs. For comparisons of ABI, ANOVA and ANCOVA were used. Three models examining the association of height with PAD and ABI were used under different adjustment schemes. The first model adjusted only age. The second model additionally adjusted for duration of diabetes, smoking status, hypertension, dyslipidemia, current medication use (antihypertensives, statins, insulin and oral antidiabetes drugs), systolic blood pressure, diastolic blood pressure, fasting plasma glucose, HbA_1c_, eGFR, albuminuria, triglyceride, total cholesterol, HDL cholesterol, LDL cholesterol and log-transformed triglyceride. The third model additionally adjusted for BMI and WHpR. *P* < 0.05 was considered to be significant. All statistical analyses were performed using SPSS statistics.

## Result

Baseline characteristics of study participants are present in Table 1. A total of 23.3 % of T2DM patients had PAD (men 22.9 % and women 23.7 %). The mean age and height were 57.8 ± 12.5 years and 170.5 cm for men, and 60.0 ± 11.7 years and 158.9 cm for women, respectively. The mean duration of type 2 diabetes was 6.7 ± 6.1 years. Among all participants, 43.3 and 38.4 % had a history of hypertension and dyslipidemia, respectively. Taller participants were more likely to be young, smoker, low BMI, low WC, low HC, and to have a short history of diabetes, hypertension, compared with those with short stature (Table 2).

**Table 1:** Clinical characteristics of subjects

	Total	Men	Women
*n*	4,528	2,397	2,131
Age (years)	59.1 (15.7)	57.8 (12.5)	60.0 (11.7)
Medical history			
Diabetes duration (years)	6.7 (6.1)	6.5 (6.0)	7.0 (6.2)
Hypertension	43.3	44.2	42.6
Dyslipidemia	38.4	39.3	37.1
Current and ex-smoker	34	60	4
Current medication use			
Antihypertensives	14.3	14.5	13.2
Statins	12.9	12.6	13.4
Insulin	33.9	33.2	35.1
Oral antidiabetes drugs	65.2	64.1	68.1
Measurements			
Height (cm)	166.0 (7.8)	170.5 (5.7)	158.9 (5.2)
Body weight (kg)	66.1 (9.3)	70.4 (7.7)	61.2 (8.7)
BMI (kg/m^2^)	24.3 (5.6)	24.4 (5.2)	24.2 (5.8)
Waist circumference (cm)	82.1 (17.3)	83.7 (16.9)	79.8 (17.7)
Hip circumference (cm)	91.2 (18.7)	90.3 (18.2)	92.7 (19.3)
Waist-to-hip ratio	0.90 (0.11)	0.93 (0.12)	0.87 (0.11)
Systolic blood pressure (mmHg)	128.9 (18.7)	128.5 (17.7)	130.1 (18.4)
Diastolic blood pressure (mmHg)	77.7 (10.2)	78.2 (10.3)	77.3 (9.6)
Fasting plasma glucose (mmol/L)	8.4 (5.4)	8.5 (5.2)	8.2 (5.7)
HbA_1c_ (%)	8.1 (2.2)	8.4 (2.3)	7.8 (2.0)
eGFR (ml/min/1.73 m^2^)	74.5 (26.1)	75.3 (23.2)	73.6 (25.4)
Albuminuria (mg g^−1^)	141 (57.4)	146 (52.5)	132 (53.9)
Triglyceride	2.2 (4.1)	2.1 (2.3)	2.1 (2.9)
Total cholesterol	4.6 (1.2)	4.5 (1.1)	4.8 (1.1)
HDL cholesterol	1.1 (0.3)	1.0 (0.2)	1.2 (0.3)
LDL cholesterol	2.6 (0.8)	2.5 (0.8)	2.6 (0.9)
ABI	1.01 (0.17)	1.02 (0.16)	1.00 (0.15)
PAD (%)	23.3	20.9	25.7

Data are means (SE) or number (%)

*NS* not significant

**Table 2:** Anthropometric and metabolic characteristics of diabetic patients stratified by sex and height quartile

	Q1	Q2	Q3	Q4	*P* value
Men					
*n*	591	610	606	590	
Height (cm)	164.2 (6.2)	168.9 (0.7)	172.3 (1.5)	178.6 (3.9)	<0.05
Age (years)	62.0 (12.0)	57.9 (11.8)	56.5 (12.8)	54.7 (12.5)	<0.05
Medical history					
Diabetes duration (years)	7.0 (6.3)	6.8 (7.0)	5.9 (5.2)	5.2 (5.2)	<0.05
Hypertension	48.9	46.5	43.1	41.6	<0.05
Dyslipidemia	36.7	39.6	40.3	39.8	NS
Current and ex-smoker (%)	51	58	61	70	<0.05
Current medication use					
Antihypertensives	14.6	14.9	14.3	14.0	NS
Statins	12.4	12.6	12.3	12.8	NS
Insulin	37.6	36.1	32.6	30.8	<0.05
Oral antidiabetes drugs	61.0	62.8	65.3	67.2	<0.05
Measurements					
Body weight (kg)	68.1 (6.3)	69.2 (7.0)	70.7 (6.8)	73.4 (9.3)	<0.05
BMI (kg/m^2^)	25.5 (5.8)	24.2 (6.0)	23.8 (5.7)	23.2 (5.4)	<0.05
Waist circumference (cm)	86.7 (17.2)	85.1 (17.0)	83.2 (16.8)	81.6 (16.9)	<0.05
Hip circumference (cm)	92.1 (18.2)	91.0 (18.6)	90.0 (18.3)	89.3 (18.6)	<0.05
Waist-to-hip ratio	0.94 (0.15)	0.95 (0.16)	0.93 (0.15)	0.92 (0.17)	NS
Systolic blood pressure (mmHg)	128.3 (20.2)	129.6 (17.1)	126.5 (17.0)	128.6 (16.1)	NS
Diastolic blood pressure (mmHg)	77.5 (11.7)	78.6 (9.8)	77.0 (10.1)	79.5 (9.2)	NS
Fasting plasma glucose (mmol/L)	8.4 (5.1)	8.3 (5.3)	8.6 (5.6)	8.5 (5.2)	NS
HbA_1c_ (%)	8.5 (2.3)	8.3 (2.1)	8.5 (2.4)	8.4 (2.3)	NS
eGFR (ml/min/1.73 m^2^)	74.9 (22.6)	76.1 (24.3)	75.6 (21.7)	75.4 (22.5)	NS
Albuminuria (mg g^−1^)	151 (48.9)	144 (53.3)	143 (51.9)	147 (50.1)	NS
Triglyceride	1.9 (2.0)	1.7 (1.3)	2.1 (2.9)	2.5 (2.8)	NS
Total cholesterol	4.5 (1.1)	4.4 (1.0)	4.6 (1.3)	4.5 (1.1)	NS
HDL cholesterol	1.0 (0.2)	1.0 (0.3)	1.0 (0.2)	1.0 (0.2)	NS
LDL cholesterol	2.5 (0.8)	2.5 (0.8)	2.8 (0.8)	2.4 (0.8)	NS
ABI	0.91 (0.01)	0.95 (0.01)	1.01 (0.01)	1.07 (0.01)	<0.05
PAD(%)	25.3	22.0	19.3	18.0	<0.05
Women					
*n*	526	546	538	521	
Height (cm)	153.1 (4.2)	158.4 (1.3)	161.4 (0.5)	167.9 (5.2)	<0.05
Age (years)	65.3 (11.4)	60.6 (11.0)	59.7 (10.8)	54.2 (11.2)	<0.05
Medical history					
Diabetes duration (years)	8.4 (6.3)	7.4 (7.0)	6.7 (5.7)	5.6 (5.2)	<0.05
Hypertension	50.3	46.3	41.2	36.3	<0.05
Dyslipidemia	37.8	36.8	36.6	37.4	NS
Current and ex-smoker (%)	6	3	5	2	<0.05
Current medication use					
Antihypertensives	14.6	13.3	12.9	12.8	NS
Statins	13.5	13.5	12.6	13.6	NS
Insulin	38.9	36.7	34.8	32.6	<0.05
Oral antidiabetes drugs	68.3	67.5	67.8	69.3	NS
Measurements					
Body weight (kg)	58.7 (8.2)	60.4 (8.4)	61.3 (7.1)	64.3 (9.8)	<0.05
BMI (kg/m^2^)	25.3 (4.9)	24.3 (5.2)	23.7 (4.8)	23.3 (4.9)	<0.05
Waist circumference (cm)	83.5 (18.2)	81.1 (17.6)	79.2 (17.9)	78.3 (18.0)	<0.05
Hip circumference (cm)	94.7 (19.3)	93.1 (19.8)	92.4 (19.4)	91.6 (19.6)	<0.05
Waist-to-hip ratio	0.88 (0.13)	0.87 (0.12)	0.86 (0.13)	0.85 (0.13)	NS
Systolic blood pressure (mmHg)	132.6 (19.7)	131.3 (19.5)	129.9 (16.4)	126.5 (17.2)	<0.05
Diastolic blood pressure (mmHg)	77.4 (10.4)	76.9 (9.5)	78.3 (9.0)	77.3 (9.5)	NS
Fasting plasma glucose (mmol/L)	7.9 (5.6)	8.3 (5.9)	8.2 (4.1)	8.3 (5.6)	NS
eGFR (ml/min/1.73 m^2^)	73.9 (23.4)	72.3 (27.1)	74.8 (28.1)	73.1 (24.6)	NS
Albuminuria (mg g^−1^)	136 (55.2)	130 (51.6)	131 (54.2)	134 (53.9)	NS
HbA_1c_ (%)	7.3 (1.7)	7.8 (2.3)	8.1 (1.9)	8.2 (2.2)	NS
Triglyceride	1.7 (1.4)	1.7 (1.1)	2.3 (2.5)	2.7 (4.3)	<0.05
Total cholesterol	4.6 (1.2)	4.7 (1.0)	4.9 (1.1)	4.9 (1.2)	NS
HDL cholesterol	1.2 (0.3)	1.2 (0.3)	1.2 (0.3)	1.1 (0.3)	NS
LDL cholesterol	2.6 (1.0)	2.6 (0.8)	2.6 (0.8)	2.6 (0.9)	NS
ABI	0.92 (0.02)	0.96 (0.01)	0.99 (0.03)	1.04 (0.02)	<0.05
PAD(%)	33.1	25.4	22.3	20.7	<0.05

Men (cm): quartile1 (Q1) 152–167; quartile2 (Q2) 168–170; quartile3 (Q3) 171–174; quartile1 (Q4) 175–188; women (cm): quartile1 (Q1) 142–156; quartile2 (Q2) 157–160; quartile3 (Q3) 161–162; quartile1 (Q4) 163–178

In both unadjusted and age-adjusted models, there were significant linear associations of height with the ABI and frequency of PAD (Table 3). An inverse association was observed between height- and gender-adjusted risk of PAD. These relationships remained unchanged following further adjustment for age, duration of diabetes, smoking status, hypertension, dyslipidemia, current medication use (antihypertensives, statins, insulin and oral antidiabetes drugs), systolic blood pressure, diastolic blood pressure, fasting plasma glucose, HbA_1c_, eGFR, albuminuria, triglyceride, total cholesterol, HDL cholesterol, LDL cholesterol and log-transformed triglyceride. Further adjustment for BMI, waist circumference, hip circumference and waist-to- hip ratio did not change the results significantly. Subjects in the shortest stature group had 1.174 times higher risk of PAD for men and 1.143 times for women, compared with those in the tallest stature group. The multivariate adjusted hazard ratios (95 % CI) of PAD for a 10-cm height increase were 0.85 (95 % CI 0.78–0.94).

**Table 3:** Logistic regression models showing associations of ABI and prevalence of PAD with quartiles of height

	Q1	Q2	Q3	Q4	*P* for trend
Men					
ABI (mean ± SE)					
Model 1	0.908 ± 0.013	0.951 ± 0.013	1.014 ± 0.013	1.065 ± 0.013	<0.001
Model 2	0.921 ± 0.012	0.954 ± 0.013	1.011 ± 0.013	1.061 ± 0.013	0.002
Model 3	0.925 ± 0.013	0.954 ± 0.013	1.006 ± 0.013	1.021 ± 0.013	0.015
Model 4	0.933 ± 0.013	0.957 ± 0.013	0.988 ± 0.013	1.010 ± 0.013	0.041
PAD					
Model 1	25.3	22.0	19.3	18.0	<0.001
Model 2	1.221 (1.062–1.337)	1.213 (1.003–1.662)	1.192 (0.863–1.779)	1.000	0.003
Model 3	1.192 (1.137–1.307)	1.131 (0.947–1.429)	1.119 (0.776–1.687)	1.000	0.021
Model 4	1.174 (1.021–1.352)	1.145 (0.957–1.372)	1.118 (0.795–1.599)	1.000	0.037
Women					
ABI (mean ± SE)					
Model 1	0.917 ± 0.012	0.956 ± 0.013	0.994 ± 0.013	1.037 ± 0.014	<0.001
Model 2	0.921 ± 0.014	0.950 ± 0.011	0.987 ± 0.014	1.031 ± 0.015	0.001
Model 3	0.926 ± 0.013	0.947 ± 0.013	0.983 ± 0.013	1.022 ± 0.013	0.016
Model 4	0.928 ± 0.013	0.945 ± 0.013	0.971 ± 0.013	1.014 ± 0.013	0.038
PAD					
Model 1	33.1	25.4	22.3	20.7	<0.001
Model 2	1.247 (1.075–1.421)	1.212 (1.011–1.879)	1.168 (0.825–2.147)	1.000	0.002
Model 3	1.243 (1.039–1.374)	1.207 (1.006–1.621)	1.116 (0.679–1.906)	1.000	0.014
Model 4	1.143 (1.054–1.569)	1.105 (1.004–1.842)	1.169 (0.762–1.976)	1.000	0.041

*Q* quartile. Q4 was the highest quartile. *Model 1* unadjusted model with height. *Model 2* adjusted for age. *Model 3* adjusted for age, duration of diabetes, smoking status, hypertension, dyslipidemia, current medication use (antihypertensives, statins, insulin and oral antidiabetes drugs),systolic blood pressure, diastolic blood pressure, fasting plasma glucose, HbA_1c_, eGFR, albuminuria, triglyceride, total cholesterol, HDL cholesterol, LDL cholesterol and log-transformed triglyceride. *Model 4* like model 3 and additionally adjusted for BMI, waist circumference, hip circumference and waist-to-hip ratio

## Discussion

This study revealed significantly negative associations between height and PAD among type 2 diabetic patients. Shorter participants with diabetes were more likely to accumulate their risks of PAD in their adulthood, compared to taller participants. In the present study, adjustment for potential risk factors did not attenuate the impact of height.

Atherosclerosis is a generalized process affecting all segments of the vascular system. The meta-analysis from 121 prospective cohort studies demonstrated that taller people have a lower risk of death from coronary disease and stroke subtypes, and show that adult short stature poses 1.5 times higher risk for coronary heart disease (CHD) morbidity and mortality than being a tall individual [9]. Some studies suggest a 10-cm increment in height to be associated with a 12–25 % reduction in risk of CHD and 20–24 % reduction in risk of stroke [10, 11]. In this study taller persons have more favorable central hemodynamics which may contribute to the inverse association between height and cardiovascular mortality. Independent of classical cardiovascular risk factors, height was found to be inversely associated with carotid atherosclerosis for overweight men [12]. PAD is the third leading cause of atherosclerotic cardiovascular morbidity, following coronary artery disease and stroke. Peripheral, coronary, and carotid manifestations of atherosclerosis strongly overlap. In our study, we found that subjects with T2DM in the shortest stature group had 1.174 times higher risk of PAD for men and 1.143 times for women, compared with those in the tallest stature group. The multivariate adjusted hazard ratios (95 % CI) of PAD for a 10-cm height increase were 0.85 (95 % CI 0.78–0.94).

The mechanism whereby height exerts negative effects on PAD in diabetic patients is not clear. While undoubtedly under a large degree of genetic control, height is influenced by early life environmental factors, which include nutrition, psychosocial stress, chronic illness and living circumstances. Height and atherosclerosis risk factors such as obesity are determined by genetic and early environmental influences. Malnutrition in childhood could be associated with short stature and poor health outcome [13, 14]. Previous studies reported inverse associations between total protein intake and diastolic blood pressure and between animal protein intake and systolic blood pressure. Hormonal factors relevant to growth, the intrauterine environment and childhood nutrition have been suggested as a potential pathway linking impaired peripheral growth to the risk of atherosclerosis in adulthood [15, 16].

It has been suggested that height may act as a crude anthropometric marker of IGF-I exposure in earlier life. The highest IGF-I levels were seen among tall adults with good linear growth [17]. IGF-1 levels were reported to be positively associated with height and inversely with risk of atherosclerosis [18]. In addition to acute metabolic insulin-like actions, IGF-I may mediate the development of atherosclerosis directly through its effects on nitric oxide production, vascular smooth muscle cell migration and proliferation and monocyte behavior and function [19].

Reduced number of nephrons in kidneys could be a possible explanation for the association between height and PAD. Birth weight and height are significantly correlated and low birth weight is known to be associated with renal disease in adults. This could be due to reduced nephron numbers, which might be reflected in lower kidney volumes, at least early in life [20–22]. Persons with reduced number of nephrons are thought to be susceptible to developing hypertension, because pressure natriuresis curves are shifted and an elevation of blood pressure is required to maintain a balance between normal sodium intake and excretion [23, 24]. The observation that taller persons have more favorable central hemodynamics may contribute to the inverse association between height and PAD [5].

The main limitation of this study concerns its observational design, so that no cause and effect relation can be deduced. Future long-term longitudinal studies will be needed to elucidate these issues. Furthermore, we did not have any information on socioeconomic status of the participants in this study; we could not adjust for socioeconomic status at baseline. The study has been conducted on type 2 diabetes patients in China and further studies should be conducted to determine whether our findings are applicable to other populations. However, despite these limitations we were able to recruit a large sample of patients with type 2 diabetes. All height, weight, waist and hip circumferences in this large study population were measured by trained personnel.

In conclusion, height is a risk factor for PAD among T2DM patients. This effect appears to be independent of other known risk factors. Although the underlying mechanism could not be identified, it may be wise to pay attention to the early detection of PAD in shorter patients with T2DM.
